# ATPase Inhibitory Factor 1 Promotes Hepatocellular Carcinoma Progression After Insufficient Radiofrequency Ablation, and Attenuates Cell Sensitivity to Sorafenib Therapy

**DOI:** 10.3389/fonc.2020.01080

**Published:** 2020-06-25

**Authors:** Jian Kong, Changyu Yao, Xuemei Ding, Shuying Dong, Shilun Wu, Wenbing Sun, Lemin Zheng

**Affiliations:** ^1^Department of Hepatobiliary Surgery, Beijing Chaoyang Hospital, Capital Medical University, Beijing, China; ^2^Key Laboratory of Molecular Cardiovascular Science of Ministry of Education, Key Laboratory of Cardiovascular Molecular Biology and Regulatory Peptides of Ministry of Health, Beijing Key Laboratory of Cardiovascular Receptors Research, School of Basic Medical Sciences, The Institute of Cardiovascular Sciences and Institute of Systems Biomedicine, Peking University Health Science Center, Beijing, China; ^3^China National Clinical Research Center for Neurological Diseases, Tiantan Hospital, Advanced Innovation Center for Human Brain Protection, Capital Medical University, Beijing, China

**Keywords:** radiofrequency ablation, hepatocellular carcinoma, ATPase inhibitory factor 1, sorafenib, epithelial-mesenchymal transition, angiogenesis

## Abstract

Epithelial-mesenchymal transition (EMT) and angiogenesis is involved in tumor progression after radiofrequency ablation (RFA). ATPase inhibitory factor 1 (IF1) is a bad predictor of prognosis. Sorafenib inhibited EMT of hepatocellular carcinoma (HCC) after RFA. Whether IF1 promotes the EMT and angiogenesis of HCC and attenuates the effect of sorafenib after insufficient RFA is investigated. In this study, higher expression of IF1 was found in residual tumor after insufficient RFA. Hep3B or Huh7 cells after insufficient RFA were designated as Hep3B-H or Huh7-H cells *in vitro*. Hep3B-H or Huh7-H cells exhibited enhanced capacities of colony formation, migration, and increased expression of EMT associated markers and IF1 compared with Hep3B or Huh7 cells. IF1 knockdown in Hep3B-H or Huh7-H cells decreased the colony formation and migratory capacity, and IF1 overexpression in Hep3B or Huh7 cells increased these capacities. IF1 in HCC cells directly and indirectly affected angiogenesis of TAECs after insufficient RFA. IF1 promoted HCC cells growth and metastasis after insufficient RFA. IF1 increased HCC cells resistance after insufficient RFA to sorafenib. Higher IF1 expression indicated poor disease survival in HCC patients after sorafenib therapy. NF-κB activation induced by IF1 attenuated the effect of sorafenib on HCC cells after insufficient RFA. Our results demonstrated that IF1 promotes the EMT and angiogenesis, and attenuates HCC cell sensitivity to sorafenib after insufficient RFA through NF-κB signal pathway.

## Introduction

Hepatocellular carcinoma (HCC) is the sixth most common neoplasm and the third leading cause of cancer death (1). Surgical resection and radiofrequency ablation (RFA) is widely accepted option for early-stage HCC patients (2, 3). Nevertheless, patients with RFA are associated with higher recurrence than surgical resection (4–6). Moreover, suboptimal RFA has been regarded as a risk factor of early diffuse recurrence for HCC (7).

Previous studies have elaborated the mechanism involving in tumor progression in HCC after RFA. Epithelial-mesenchymal transition (EMT) is involved in the above procedure (8–14). Sublethal heat treatment facilitated EMT and promoted the malignant potential of HCC (10, 15). Incomplete RFA accelerated invasiveness and metastasis of residual cancer of HCC cells through β-catenin signal pathway (11). Akt and ERK signaling pathways also participated in the process of EMT in HCC after insufficient RFA (8). LncRNA FUNDC2P4 down-regulation promoted EMT through decreasing E-cadherin expression in residual HCC after insufficient RFA (13). EMT-related genes were associated with aggressive local recurrence of HCC after RFA (9). Tumor associated endothelial cells (TAECs) also showed enhanced angiogenesis and facilitated invasiveness of residual HCC after insufficient RFA (16). We previously showed that sorafenib could inhibit EMT of HCC after RFA (17). However, the mechanism involved in the process is still unclear.

H+-ATP synthase in the mitochondrial, which is known as F0F1ATPase or complex V mainly, regulates energy metabolism (18). ATPase inhibitory factor 1 (IF1) is involved in regulating energy metabolism through directly binding with the βF1subunit and inhibits ATP hydrolysis (19). A high expression level of IF1 in the tumor indicates poor survival and recurrence of liver, lung, gastric, and glioma cancer patients (20–23). IF1 increased vascular endothelial growth factor and snail expression by activating NF-κB signaling, which depended on the binding of TRAF1 to NIK (23). Previous study showed that IF1 upregulation promoted tumor cells survival under temporary hypoxic environment through reserving cellular ATP despite mitochondria dysfunction (24). RFA could lead to a transition zone formation between necrotic coagulation and normal liver tissues, where blood coagulation and thrombosis exposed residual tumor cells to a hypoxic environment (25). Hypoxia may promote the progression of residual tumor, in which HIF-1α was involved (26–28). We also previously demonstrated that HIF-1α inhibitor, YC-1 could enhance anti-tumor activity of sorafenib in HCC (29). Therefore, we assumed that IF1 played the role in the EMT and angiogenesis of HCC and was involved in the effect of sorafenib after insufficient RFA.

In this study, we showed that IF1 participated in the EMT and angiogenesis of HCC after insufficient RFA. Ectopic overexpression of IF1 attenuated the suppressing effect of sorafenib on EMT and angiogenesis in HCC after RFA, and higher expression of IF1 in HCC tissue indicated low disease free survival for HCC patients receiving resection and sorafenib. Collectively, Our results provide an explanation for progression of HCC after insufficient RFA.

## Materials and Methods

### Reagents and Antibodies

Sorafenib was obtained from Bayer Pharmaceuticals. Antibodies of anti-IF1, anti-N-cadherin, anti-snail, and anti-Vimentin were bought from Cell Signaling Technology (Beverly, CA, USA). Antibodies of anti-CD31 and anti-PCNA were obtained from ZSGB-BIO (Beijing, China). Anti-β-actin antibody was bought from Beyotime (Jiangsu, China). Lipo2000 was bought from Thermo Fisher (Waltham, MA, USA).

### Cell Culture

Hep3B and Huh7 cells were grown in high-glucose Dulbecco's modified Eagle medium (DMEM) at 37°C in a humidified incubator with 5% CO_2_.

### TAECs Isolation

TAECs were isolated as described before (16). TAECs were grown in complete ECM medium (Sciencell) and used at passages 1–6.

### Heat Treatment *in vitro*

Insufficient RFA was simulated *in vitro* as described before (8). Briefly, HCC cells were seeded into the 6-well-plates and the plates were sealed and submerged in a water bath set to 47°C for 5 min. Thereafter, cells were allowed to recover, and when the surviving populations reached 80% confluence, cells were propagated into the 6-well-plates and exposed to above heat treatment for 10 min. Then the process was repeated and cells were sequentially exposed to above-mentioned treatment for 15, 20, and 25 min. Survived cells from the heat treatment for 25 min were designated as Hep3B-H and Huh7-H cells.

### Colony Formation Assay

Colony formation assay was performed as described before (29).

### Wound-Healing Assay

The 5 × 10^5^ cells were cultured in 6-well-plates until 90% confluent. A sterile yellow pipette tip was employed to make a straight scratch. The suspension cells were washed off thrice gently. Then, the medium was replaced and images of the same location were observed for next days. Crystal violet was used to stain the cells (Beyotime, Nantong, China) and photographed.

### Transwell Assay

Transwell assay was performed using a modified Boyden chamber (Costar-Corning, New York, USA) with 8.0-μm pore polycarbonate filter inserts in 24-well-plates as described before (8).

### Lactate Measurement

Lactate concentration in HCC cells were examined using lactate assay kit (Solarbio, Beijing, China) according to the procedure.

### Western Blot Analysis

HCC cells were lysed with RIPA lysis buffer (Solarbio, Beijing, China) containing protease and phosphatase inhibitor. The concentration of cell lysate protein was determined using a Bicinchoninic acid (BCA) protein assay kit. The following process was performed as described before (29).

### Dual-Luciferase Reporter Assay

HCC cell lines (3 × 10^5^/well) in 12-well-plates were transfected with reporter plasmids encoding pNF-κB-luc (60 ng) and pEF-Renilla-luc (10 ng) using Lipofectamine 2000. After 4 h, the medium was replaced and sorafenib was added. After 24 h, cell lysates were prepared, and luciferase activity was measured using a Dual-Luciferase Assay Kit (Promega, Madison, WI, USA).

### Animals

Male BALB/c nu/nu mice (4–6 weeks of age) were obtained from Vital River laboratories (Beijing, China) and housed under defined flora conditions in individually ventilated sterile micro-isolator cages. All experimental procedures were approved by the Animal Care and Use Committee of Capital Medical University (Beijing, China).

### Insufficient RFA *in vivo*

In the orthotopic model, Hep3B cells (5 × 10^6^) were suspended in 200 μl serum-free DMEM and then implanted subcutaneously into the upper right flank region of nude mice. When the tumor reached approximately 1 cm in length, it was removed, minced into small pieces of equal volume (2 × 2 × 2 mm^3^), and transplanted into the livers of nude mice (*n* = 5 per group). The tumors were ablated with insufficient RFA. A radiofrequency current generator (cool-tip RFA generator, Covidien, Mansfield, MA, USA) was used to generate radiofrequency energy. To deliver the radiofrequency energy, we used a 17-gauge cool-tip electrode of 15 cm length with 0.7 cm exposed tip (Covidien, Mansfield, MA, USA). Each ablation cycle lasted for 10 s. The animals were sacrificed 3 weeks after insufficient RFA.

### Tail Vein Metastatic Assay and Ectopic HCC Model

Tail vein metastatic assay (*n* = 5 or 6 per group) and ectopic HCC model (*n* = 6 per group) was performed as described before (8). The animals were sacrificed 4 weeks after tumor cells implantation or sorafenib treatment.

### *In vivo* Matrigel Plug Angiogenesis Assay

Briefly, 1 × 10^6^ TAECs premixed with (Matrigel growth factor-reduced) were subcutaneously implanted into the flanks of the nude mouse (*n* = 3 per group). After 2 weeks, the animals were sacrificed, and the plugs were collected.

### Immunofluorescence

HCC cells were seeded on coverslips and cultured for 24 h. Cells were washed twice with PBS, fixed with 4% paraformaldehyde for 20 min and blocked with 5% BSA for 1 h. Then cells were incubated with anti-N-cadherin antibody for 90 min at 37°C, followed by with fluorescence labeled secondary antibody for 30 min at room temperature. DAPI was used to visualize nuclei. The stained cells were observed under laser scan confocal microscopy.

### Immunohistochemistry

The procedures of immunocytochemistry were described in the previous study (29).

### Ectopic Expression and Knockdown of IF1

Lentiviral vectors, which encoded the human IF1 gene or shRNA-IF1 were constructed in LiKeli BioTECH Co. Ltd. (Beijing, China). The IF1 shRNA sequence was as follows: 5′-CACCATGAAGAAGAAATCGTT-3′. The empty vectors were used as negative control. The lentiviral vectors were transfected into Hep3B and Huh7 cells with a multiplicity of infection (MOI) of 20 to 30 in the presence of polybrene (2 μg/ml). Transfected cells were selected for 2 weeks with 2 μg/ml puromycin after 48 h. Pooled populations of knockdown cells and overexpression cells, which were acquired 2 weeks after drug selection without subcloning, were used in the experiments.

### CM Collection

HCC cells were cultured in serum-free DMEM, and after 24 h the CM was collected. The medium was centrifuged and stored at −80°C until needed.

### Tube Formation Assay

Growth factor-reduced Matrigel (10 mg/ml; BD Biosciences) was thawed overnight at 4°C, and 70 μl was added to each well of a 96-well-plate and allowed to solidify for 30 min at 37°C. Wells were incubated with 1 × 10^4^ TAECs cells or Mixed 1 × 10^4^ TAECs with 1 × 10^4^ HCC cells for 4 h. Capillary tube formation was observed and total length and number of junctions of the tubes were quantified using ImageJ software and the Angiogenesis Analyzer plugin of the capillary-like structures.

### Clinical Samples

Human tissue microarray contained 59 cases of HCC samples and corresponding non-cancerous liver tissues after sorafenib therapy was bought from Superbiotek (Shanghai, China).

### Statistical Analysis

All values are expressed as the mean ± SD. The data were analyzed using Student's *t*-test or the ANOVA test. Kaplan-Meier analyses were used for survival analysis. A *P* < 0.05 was considered statistically significant. GraphPad Prism (GraphPad Software Inc., San Diego, California, USA) was used for these analyses.

## Results

### Higher Expression of IF1 Is Found in Orthotopic Xenograft HCC Model After Insufficient RFA

**Figure 1:**
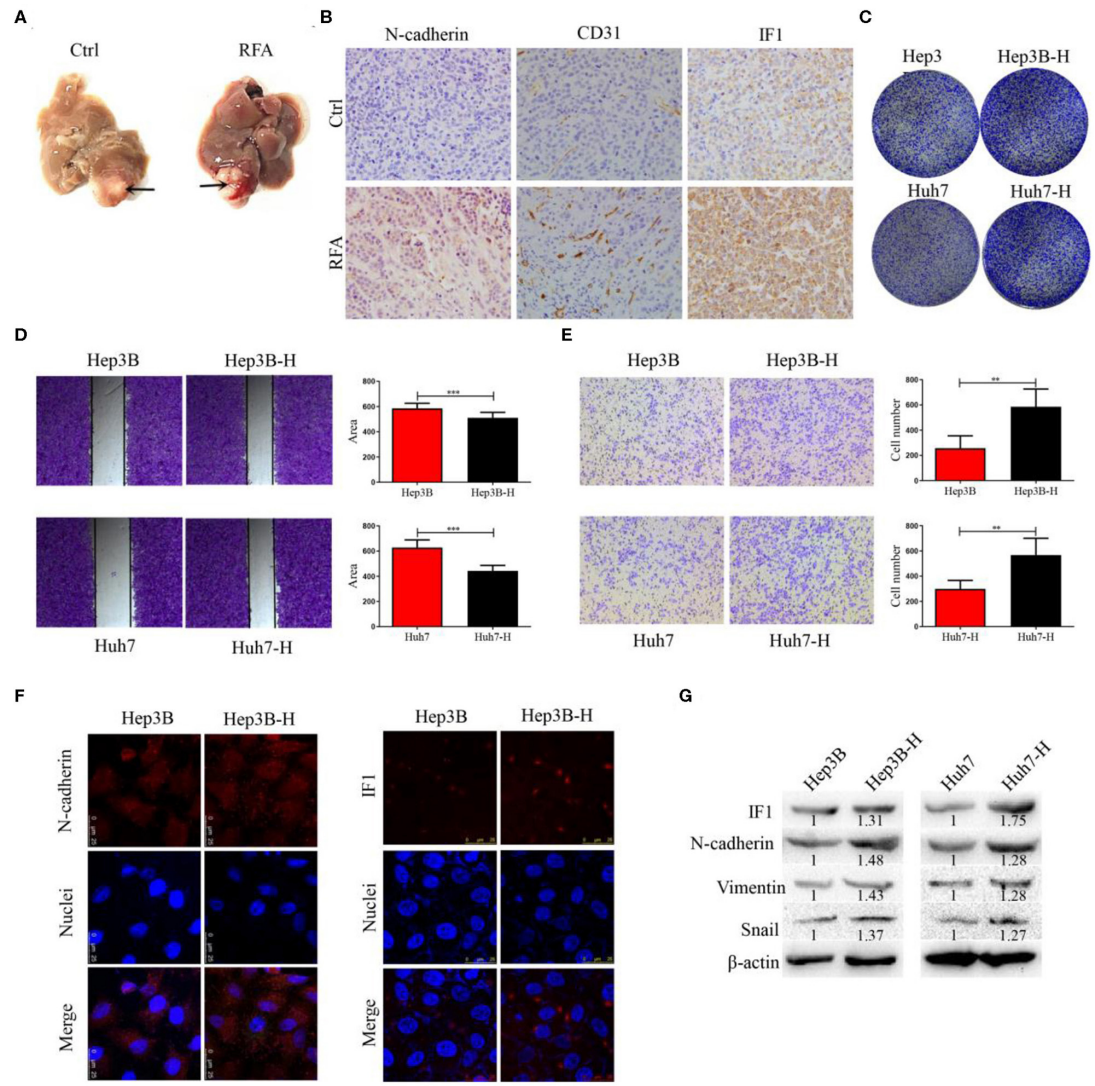
The expression of IF1 in orthotopic xenograft HCC model after insufficient RFA. **(A)** Orthotopic xenograft tumor was treated with insufficient RFA and residual tumor was collected (*n* = 5 per group). After 3 weeks, the animals were sacrificed. **(B)** The expression of IF1, CD31, and N-cadherin were shown. Hep3B and Huh7 cells were treated with heat treatment and survived cells were designated as Hep3B-H and Huh7-H, respectively. Hep3B-H or Huh7-H cells showed increased colony formation capacities **(C)** and migration **(D,E)** compared with Hep3B or Huh7 cells, respectively. **(F,G)** EMT-related markers and transcription factor was tested by immunofluorescence assay and Western blot. The expression of IF1 in Hep3B-H or Huh7-H cells was shown. Data are the means ± SD of at least three experiments.^**^P < 0.01, ^***^P < 0.001.

Orthotopic xenograft tumor model was established to explore the effect of insufficient RFA on residual tumor progression. The orthotopic tumor was treated with insufficient RFA and residual tumors were collected (Figure 1A). The expression of IF1, CD31, and N-cadherin was higher in residual tumors than those without treatment (Figure 1B). Hep3B and Huh7 cells survived from the heat treatment were designated as Hep3B-H and Huh7-H, respectively. Hep3B-H or Huh7-H cells demonstrated increased colony formation capacities in comparison with Hep3B or Huh7 cells, respectively (Figure 1C), which suggested that the proliferation of Hep3B-H and Huh7-H cells were increased. Meanwhile, Hep3B-H or Huh7-H cells showed a 16.7 or 20.1% increased proliferation ability compared with Hep3B or Huh7 cells, respectively at 72 h (Figure S1). The wound-healing assay and transwell assay were performed to evaluate HCC cells migration. Hep3B or Huh7 cells underwent a slower closing of scratch wound compared with Hep3B-H or Huh7-H cells, respectively (Figure 1D). Similarly, a significantly increased numbers of Hep3B-H or Huh7-H cells were observed on the outside membranes of the transwell chamber compared with Hep3B or Huh7 cells, respectively (Figure 1E). To investigate whether EMT occur in Hep3B-H and Huh7-H cells, markers and transcription factors associated with EMT was tested by immunofluorescence assay and Western blot. Hep3B-H cells or Huh7-H showed higher expression of N-cadherin compared with Hep3B or Huh7 cells (Figure 1F, Figure S2), which suggested epithelial cells returned to mesenchymal cells. As expected, elevated N-cadherin, snail, and Vimentin expression was observed in Hep3B-H and Huh7-H cells (Figure 1G). Meanwhile, Hep3B-H and Huh7-H cells showed increased IF1 expression (Figures 1F,G, Figure S2).

### IF1 Promotes EMT, Cell Proliferation, and Migration in HCC Cells After Insufficient RFA

**Figure 2:**
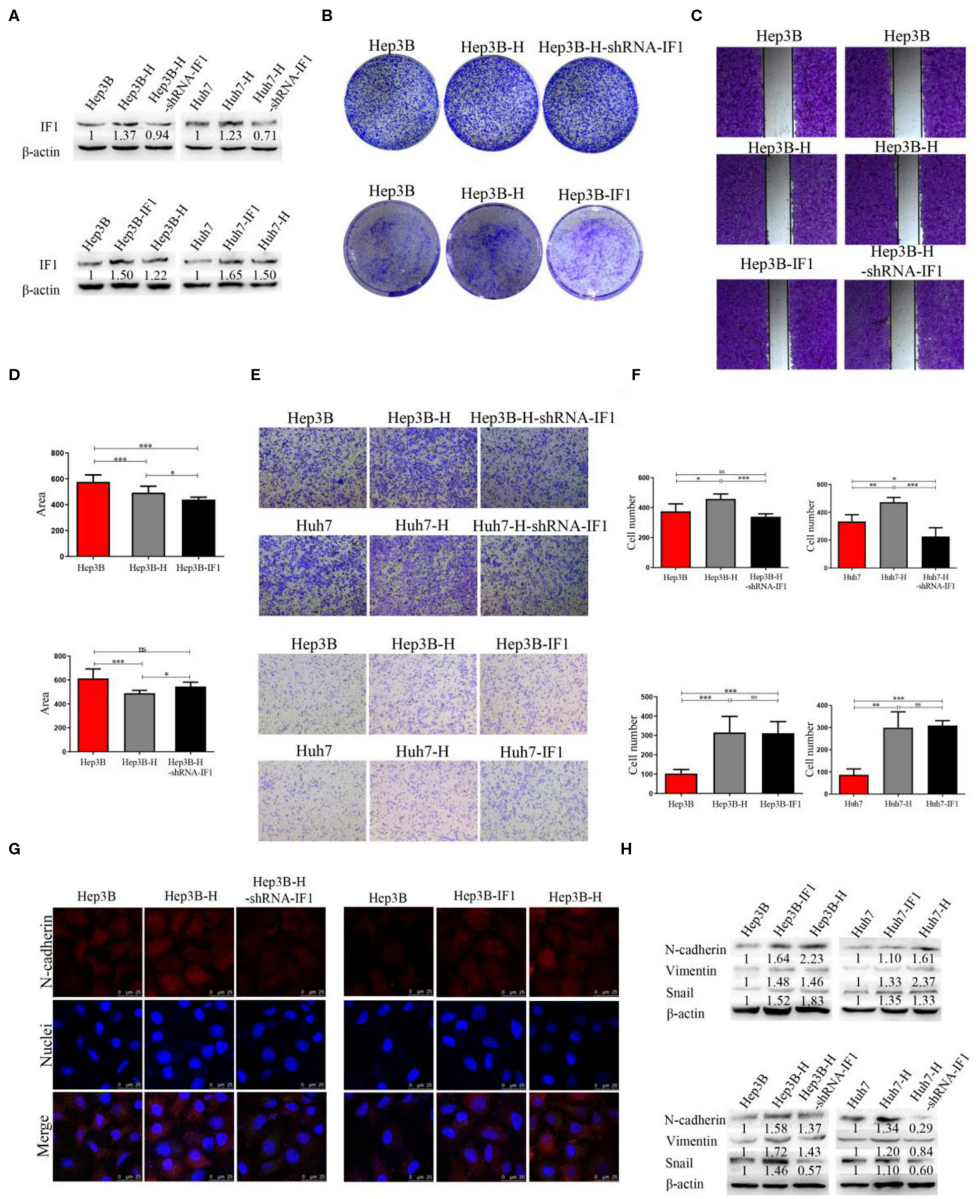
IF1 promoted EMT, cell proliferation, and migration in HCC cells after insufficient RFA. IF1 was overexpressed in Hep3B and Huh7 cells (Hep3B-IF1 and Huh7-IF1) and knocked down in Hep3B-H and Huh7-H cells (Hep3B-H-shRNA-IF1) and Huh7-H-shRNA-IF1. **(A)** Western blot validation of the transfection efficiency in Hep3B or Huh7 cells. **(B)** Colony formation assay analysis of cell proliferation after IF1 knockdown or overexpression in Hep3B cells after insufficient RFA. **(C,D)** Wound healing assay analysis of cell migration after IF1 knockdown or overexpression in Hep3B cells after insufficient RFA. **(E,F)** Transwell assay analysis of cell migration after IF1 knockdown or overexpression in HCC cells after insufficient RFA. **(G)** Immunofluorescence analysis of the N-cadherin and IF1 expression after IF1 knockdown or overexpression in Hep3B cells after insufficient RFA. **(H)** Western blot analysis of N-cadherin, snail, and Vimentin expression after IF1 knockdown or overexpression in HCC cells after insufficient RFA. Data are the means ± SD of at least three experiments. ^*^P < 0.05, ^**^P < 0.01, ^***^P < 0.001; ns, no significance.

To verify whether IF1 displayed a specific role in EMT of HCC after insufficient RFA, Hep3B, Hep3B-H, Huh7, and Huh7-H cells was used to construct IF1 overexpressed and knocked down cell lines for further phenotype and function studies. IF1 was overexpressed in Hep3B and Huh7 cells (Hep3B-IF1 and Huh7-IF1), and knocked down in Hep3B-H and Huh7-H cells (Hep3B-H-shRNA-IF1 and Huh7-H-shRNA-IF1). IF1 expression was examined by western blot (Figure 2A). Compared with Hep3B and Huh7 cells, Hep3B-H and Huh7-H cells showed enhanced capacity of colony formation (Figure 2B, Figure S3A). However, Hep3B-H-shRNA-IF1 and Huh7-H-shRNA-IF1 cells showed impaired capacity of colony formation compared with Hep3B-H and Huh7-H cells, and Hep3B-IF1 and Huh7-IF1 cells showed enhanced capacity of colony formation compared with Hep3B and Huh7 cells (Figure 2B, Figure S3A). Furthermore, we assessed the influence of IF1 on migratory abilities in HCC after insufficient RFA. The migratory capacity in Hep3B-H cells was increased compared with that in Hep3B cells, but IF1 knockdown in Hep3B-H cells decrease the migratory (Figures 2C–F). IF1 overexpression in Hep3B showed increased migratory. And, Hep3B and Hep3B-H-shRNA-IF1 or Hep3B-IF1 and Hep3B-H exhibited no difference influenced (Figures 2C–F). Similar results were also found in Huh7 cells (Figures 2E,F). We also investigated whether IF1 influenced the EMT of HCC after insufficient RFA. Hep3B-H cells showed higher expression of N-cadherin compared with Hep3B cells, but IF1 knockdown in Hep3B-H cells decreased the expression of N-cadherin and IF1 overexpression in Hep3B cells increased the expression of N-cadherin (Figure 2G). Decreased N-cadherin, snail, and Vimentin expression and increased E-cadherin expression was also found in Hep3B-H-shRNA-IF1 or Huh7-H-shRNA-IF1cells compared with Hep3B-H or Huh7-H cells (Figure 2H, Figure S4). Furthermore, an increased lactate concentration were found in Hep3B-H or Huh7-H cells compared with Hep3B or Huh7, respectively (Figure S5). Meanwhile, IF1 knockdown decreased the lactate concentration in Hep3B-H or Huh7-H cells, and IF1 overexpression increased the lactate concentration in Hep3B or Huh7 cells (Figure S5). Our results indicated an enhanced glycolysis activity in HCC after insufficient RFA.

### IF1 in HCC Cells Directly and Indirectly Facilitates Angiogenesis of TAECs After Insufficient RFA

**Figure 3:**
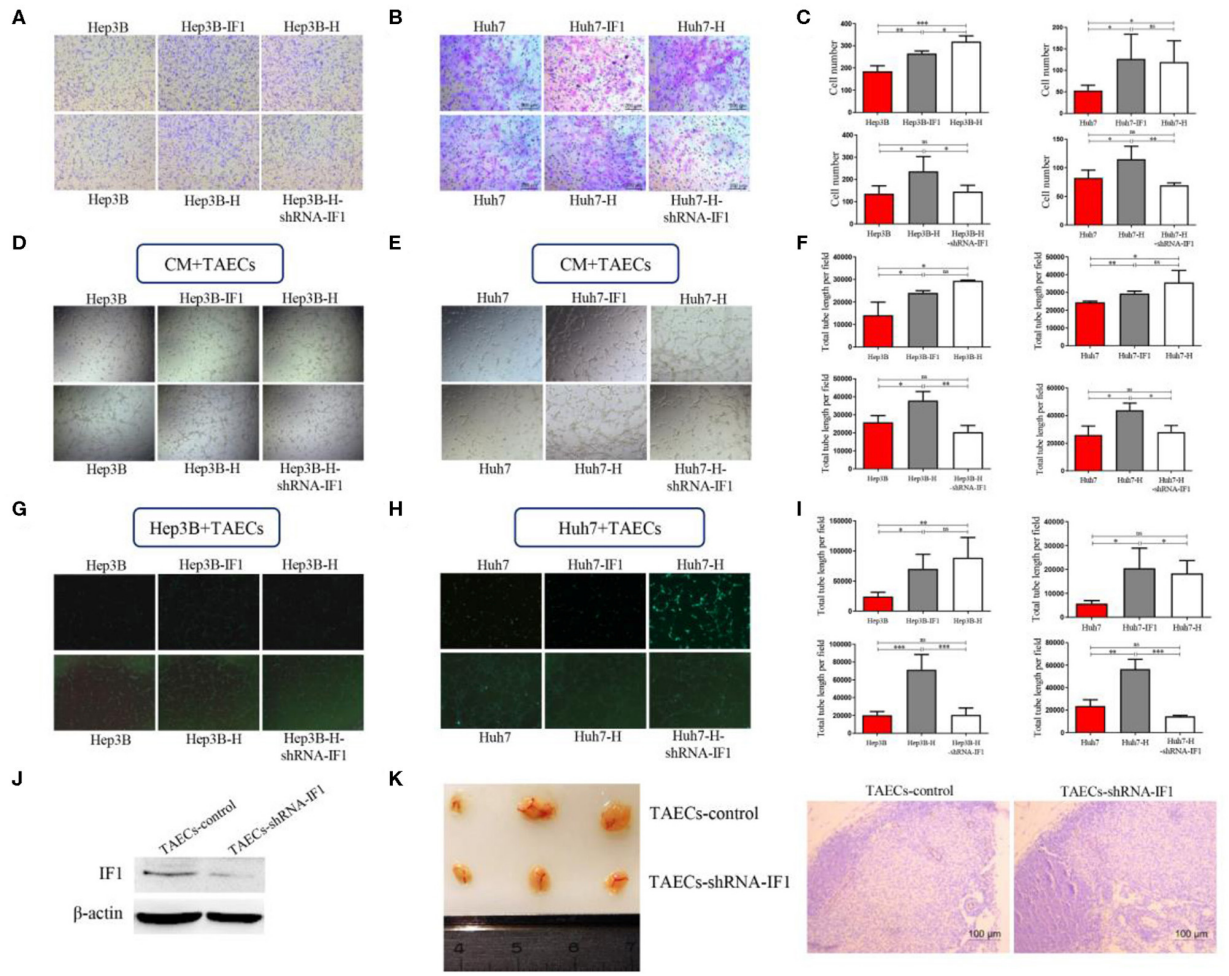
IF1 in HCC cells indirectly and directly affected angiogenesis of TAECs after insufficient RFA. CM from HCC cells after IF1 knockdown or overexpression with or without insufficient RFA was collected. **(A,B)** Transwell assay analysis of TAECs migration after co-culture with CM from Hep3B and Huh7 cells. **(C)** The statistical results of **(A,B)** were shown. **(D,E)** Analysis of TAECs tube formation after co-culture with CM from Hep3B and Huh7 cells. **(F)** The statistical results of **(D,E)** were shown. **(G,H)** Hep3B or Huh7 cells (with GFP) were co-cultured with TAECs and VM was observed. **(I)** The statistical results of **(G,H)** were shown. **(J)** Western blot validation of the transfection efficiency in TAECs. **(K)** The matrigel plug assay containing TAECs-shRNA-IF1 and control was performed. Data are the means ± SD of at least three experiments. *P < 0.05, **P < 0.01, ***P < 0.001; ns, no significance.

To determine whether IF1 influenced the angiogenesis of TAECs after insufficient RFA, we established a co-culture system using HCC cells and TAECs. We collected CM from HCC cells in 4 groups (Hep3B, Hep3B-H, Hep3B-H-shRNA-IF1; Hep3B, Hep3B-IF1, Hep3B-H; Huh7, Huh7-H, Huh7-H-shRNA-IF1; Huh7, Huh7-IF1, Huh7-H). We then cultured TAECs with these media and evaluated migration and tube formation of TAECs. The CM of Hep3B-H or Huh7-H was more potent in promoting TAECs migration (Figures 3A–C) and tube formation (Figures 3D–F) compared with those from Hep3B or Huh7, respectively. However, the CM of Hep3B-H-shRNA-IF1 or Huh7-H-shRNA-IF1 was less effective in stimulating TAECs migration (Figures 3A–C) and tube formation (Figures 3D–F) than those from Hep3B-H or Huh7-H. The CM of Hep3B-IF1 or Huh7-IF1 was more potent in inducing TAECs migration (Figures 3A–C) and tube formation (Figures 3D–F) compared with Hep3B and Huh7 cells. Moreover, CM from Huh7-IF1 and Huh7-H showed no difference, but CM from Hep3B-IF1 and Hep3B-H exhibited significant difference (Figures 3A–F). Similarly, CM from (Hep3B and Hep3B-H-shRNA-IF1) or (Huh7 and Huh7-H-shRNA-IF1) also showed no difference (Figures 3A–F).

To further observe the direct effect of IF1 on interaction between HCC cells and TAECs after insufficient RFA, mixed TAECs, and HCC cells (with GFP) were added to Matrigel to study the tube formation. We found that HCC cells could form VM when TAECs were present. Hep3B-H or Huh7-H cells were more potent to form VM compared with Hep3B or Huh7 cells (Figures 3G–I). However, IF1 knockdown in Hep3B-H or Huh7-H cells could decrease the capacity of VM and IF1 overexpression in Hep3B or Huh7 cells could enhance the capacity of VM (Figures 3G–I). Furthermore, (Hep3B and Hep3B-H-shRNA-IF1) or (Hep3B-IF1 and Hep3B-H) showed no difference (Figures 3G–I). Similar results were also observed in Huh7 cells (Figures 3G–I). To further confirm whether IF1 directly influenced TAECs angiogenesis, an *in vivo* matrigel plug assay was used to examize the new blood vessels formed in the transplanted matrigel plugs in mice. IF1 was knocked down in TAECs and the expression of IF1 was confirmed by western blot (Figure 3J). The plugs containing TAECs-shRNA-IF1 significantly reduced the vessel length compared with control (Figure 3K). CD31 in the plugs containing TAECs-shRNA-IF1 was lower compared with control (Figure 3K).

### IF1 Promotes Tumor Growth and Metastasis of HCC Cells After Insufficient RFA *in vivo*

**Figure 4:**
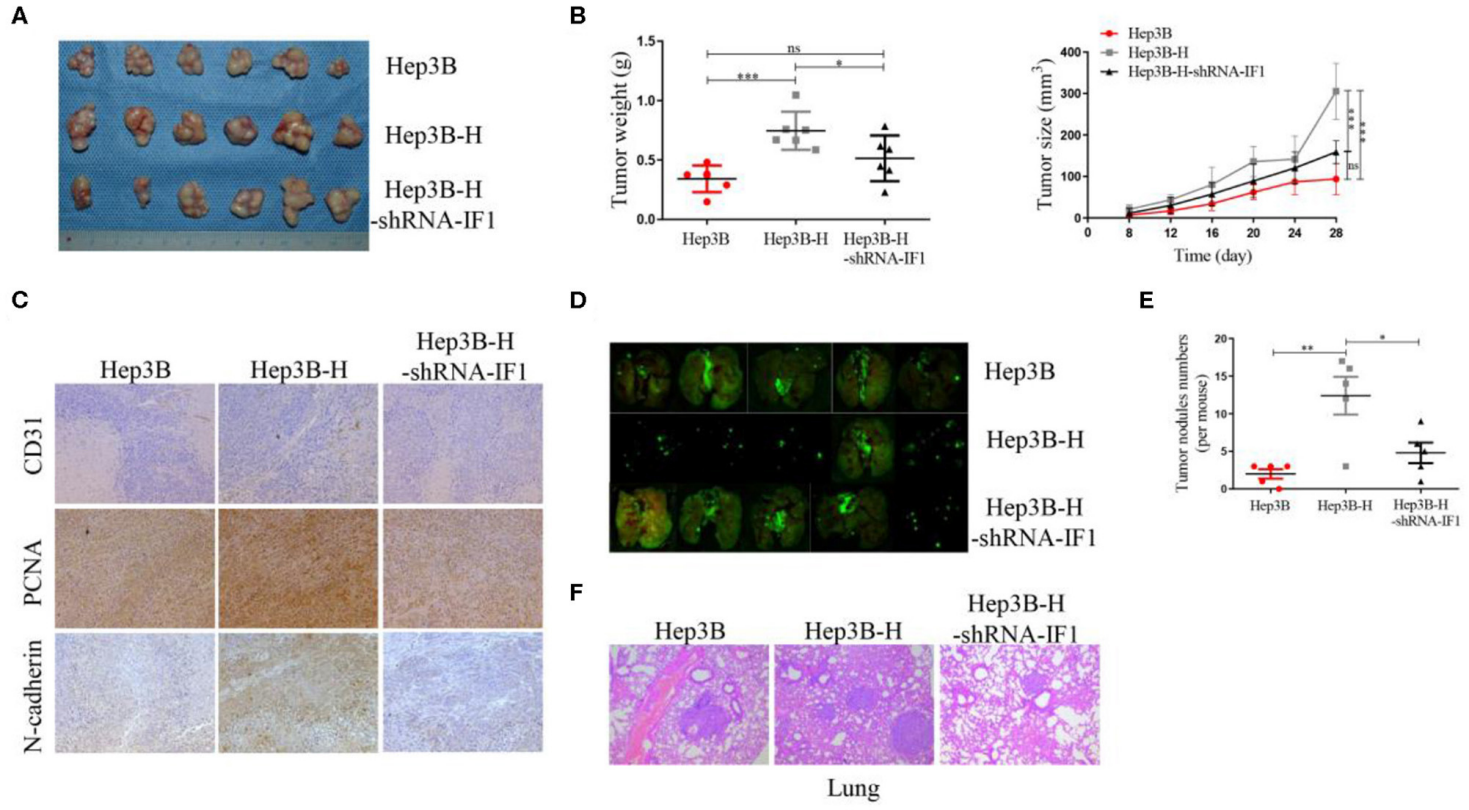
IF1 promoted tumor growth and metastasis of HCC cells after insufficient RFA *in vivo*. **(A)** Hep3B, Hep3B-H, and Hep3B-H-shRNA-IF1 cells were inoculated subcutaneously into nude mice. After 4 weeks, the animals were sacrificed. Images of tumor volume were exhibited. *n* = 6 per group. **(B)** Tumor size was measured with a caliper rule every 4 days and tumor weight was measured at the end of the experiment. **(C)** Tumor sections were stained with PCNA, N-cadherin, and CD31. Representative images of the immunohistochemistry assay were shown. **(D)** *In-vivo* Imaging System was used to quantify metastatic nodules of Hep3B, Hep3B-H, and Hep3B-H-shRNA-IF1 cells. The animals were sacrificed 4 weeks after tumor cells injection. Pictures of lung metastatic nodules were shown at the end of the experiment. *n* = 5 per group. **(E)** The number of lung metastatic nodules was counted. **(F)** The representative images of HE were shown. Data are the means ± SD. *P < 0.05, **P < 0.01, ***P < 0.001; ns, no significance.

To further determine the tumorigenesis potential of IF1 *in vivo*, Hep3B, Hep3B-H, and Hep3B-H-shRNA-IF1 cells were inoculated subcutaneously into nude mice. Hep3B-H xenograft tumors showed an increase in tumor volume and weight compared with the Hep3B xenograft tumors. Hep3B-H-shRNA-IF1 xenograft tumors showed a reduction in tumor volume and weight compared with Hep3B-H xenograft tumors (Figures 4A,B). Hep3B xenograft tumors and Hep3B-H-shRNA-IF1 xenograft tumors showed no difference (Figures 4A,B). Hep3B-H xenograft tumors expressed higher levels of N-cadherin, CD31, and PCNA relative to Hep3B xenograft tumors (Figure 4C). However, Hep3B-H-shRNA-IF1 tumors expressed lower levels of those proteins compared with Hep3B-H xenograft tumors (Figure 4C). Next, we determined the effects of IF1 on the metastasis of HCC cells after insufficient RFA by *in-vivo* Imaging System to quantify metastatic nodules. The number of metastatic nodules was increased in mice injected with Hep3B-H cells in comparison with those injected with Hep3B cells (Figures 4D,E). Inhibition of IF1 in Hep3B-H cells decrease the number of lung metastatic nodules (Figure 4E). Furthermore, the mice injected with Hep3B or Hep3B-H-shRNA-IF showed no difference (Figure 4E). The difference was further confirmed by the histopathological analysis with HE staining (Figure 4F). No apparent changes were observed in body weight in different groups (Figure S6).

### IF1 Attenuates Sensitivity of HCC Cells to Sorafenib After Insufficient RFA *in vitro*, and Indicates Poor Disease Free Survival in HCC Patients After Sorafenib Therapy

**Figure 5:**
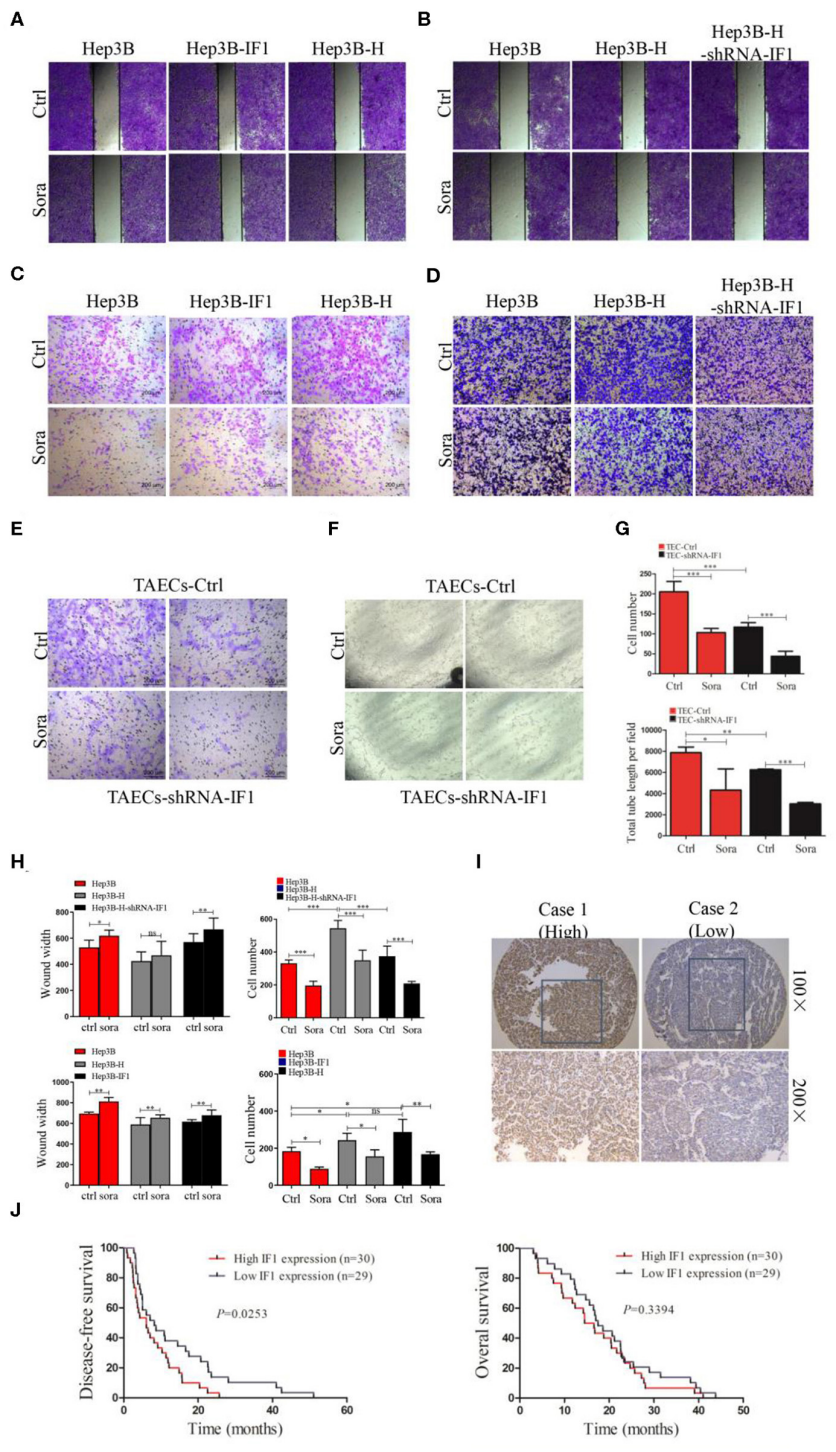
IF1 attenuated sensitivity of HCC cells after insufficient RFA to sorafenib *in vitro*. **(A,B)** The effect of sorafenib on wound-healing capacity of Hep3B, Hep3B-IF1, and Hep3B-H or Hep3B, Hep3B-H, and Hep3B-H-shRNA-IF1 was shown. **(C,D)** The effect of sorafenib on migration of Hep3B, Hep3B-IF1, and Hep3B-H or Hep3B, Hep3B-H, and Hep3B-H-shRNA-IF1 was shown. **(E,F)** The influence of sorafenib on migration and tube formation of TAECs-shRNA-IF1 and TAECs was exhibited. **(G)** The statistical results of **(F,G)** were shown. **(H)** The statistical results of **(A–D)** were shown. **(I)** The immunohistochemical staining of IF1 in HCC tissue with sorafenib therapy. **(J)** Kaplan-Meier analysis of DFS and OS for the expression IF1. Data are the means ± SD. *P < 0.05, **P < 0.01, ***P < 0.001; ns, no significance.

We further determine the effect of IF1 on sorafenib suppressing HCC cells after insufficient RFA. Sorafenib could inhibit the colony formation (Figures S3B, S7A,B) and migration (Figures 5A–D) of HCC cells. The influence of sorafenib on Hep3B-H cells was less effective compared with that on Hep3B cells, but IF1 knocked down in Hep3B-H could restore the effect of sorafenib (Figures 5A–D). Sorafenib slowed the closing of scratch wound of Hep3B, Hep3B-H, and Hep3B-H-shRNA-IF1 cells by 17, 10.3, and 17% respectively, and decreased the migratory capacity of Hep3B, Hep3B-H, and Hep3B-H-shRNA-IF1 cells by 40.8, 35.8, and 44.3%, respectively (Figure 5H). Meanwhile, IF1 overexpression in Hep3B cells could reduce the sorafenib sensitivity. Sorafenib slowed the closing of scratch wound of Hep3B, Hep3B-IF1, and Hep3B-H cells by 17, 11.1, and 10.2% respectively, and decreased the migratory capacity of Hep3B, Hep3B-IF1, and Hep3B-H cells by 51.7, 36, and 41.6%, respectively (Figure 5H). Similar results were observed in Huh7 cells (Figures S7C,D).

We also observe the effect of IF1 on sorafenib inhibiting TAECs. Sorafenib could reduce the migration and tube formation of TAECs by 49.6 and 44.9% (Figures 5E–G). However, after knocking down IF1 in TAECs, sorafenib suppressed the TAECs migration and tube formation by 62.6 and 51.6% (Figures 5E–G). Together, results from different assays in HCC cells and TAECs suggest that IF1 decreases the efficacy of sorafenib to suppress the HCC cells and TAECs.

To determine the clinical significance of IF1 in HCC after sorafenib therapy, *in situ* hybridization assay was performed in tumor tissues from 59 HCC patients. The correlations among IF1 expression levels and the clinicopathological parameters of the HCC patients were shown in Table 1. The correlations between IF1 and tumor differentiation and TNM stage were not significant (Table 1). The patients were divided into IF1 low-expressed and IF1 high-expressed group when cut off with a median value of IF1 expression. The expression of IF1 was significantly correlated to patient age (Table 1). Low disease free survival (DFS) (6.1 vs. 8.2 months, *P* = 0.0253) was found in the IF1 high-expressed group than the ones in low-expressed (Figures 5I,J). However, overall survival (OS) (15.6 vs. 17.5 months, *P* = 0.3394) in the IF1 high-expressed group and the ones in low-expressed group was not significant (Figure 5J).

**Table 1:** Correlation of the clinicopathological finding with IF1 expression in 59 HCC patients with sorafenib therapy.

**Variable**		**IF1**		***P*-value**
		**High expression**	**Low expression**	
Age (year)	≤50	14	23	0.0150^*^
	>50	16	6	
Gender	Male	29	28	1.0000
	Female	1	1	
HBs antigen	Negative	5	3	0.7065
	Positive	25	26	
Liver cirrhosis	Absence	13	15	0.6058
	Presence	17	14	
Gamma-glutamytransferase (U/L)		158.4	127.6	0.3748
Serum AFP (ng/ml)	≤20	7	1	0.0523
	>20	23	28	
ALT (U/L)		136.6	180.1	0.6654
Tumor multiplicity	Single	28	26	0.6707
	Multiple	2	3	
Tumor encapsulation	Absence	16	13	0.6058
	Presence	14	16	
Tumor differentiation	II–III	29	28	1.0000
	IV	1	1	
Vascular invasion	Absence	8	7	0.7611
	Presence	22	22	
Tumor size (cm)	≤5	12	16	0.6211
	>5	20	19	
TNM stage	I	5	4	0.6531
	II	23	21	
	III–IV	2	4	

* *P <0.05*.

### IF1 Decreases the Sensitivity of HCC Cells After Insufficient RFA to Sorafenib *in vivo*

**Figure 6:**
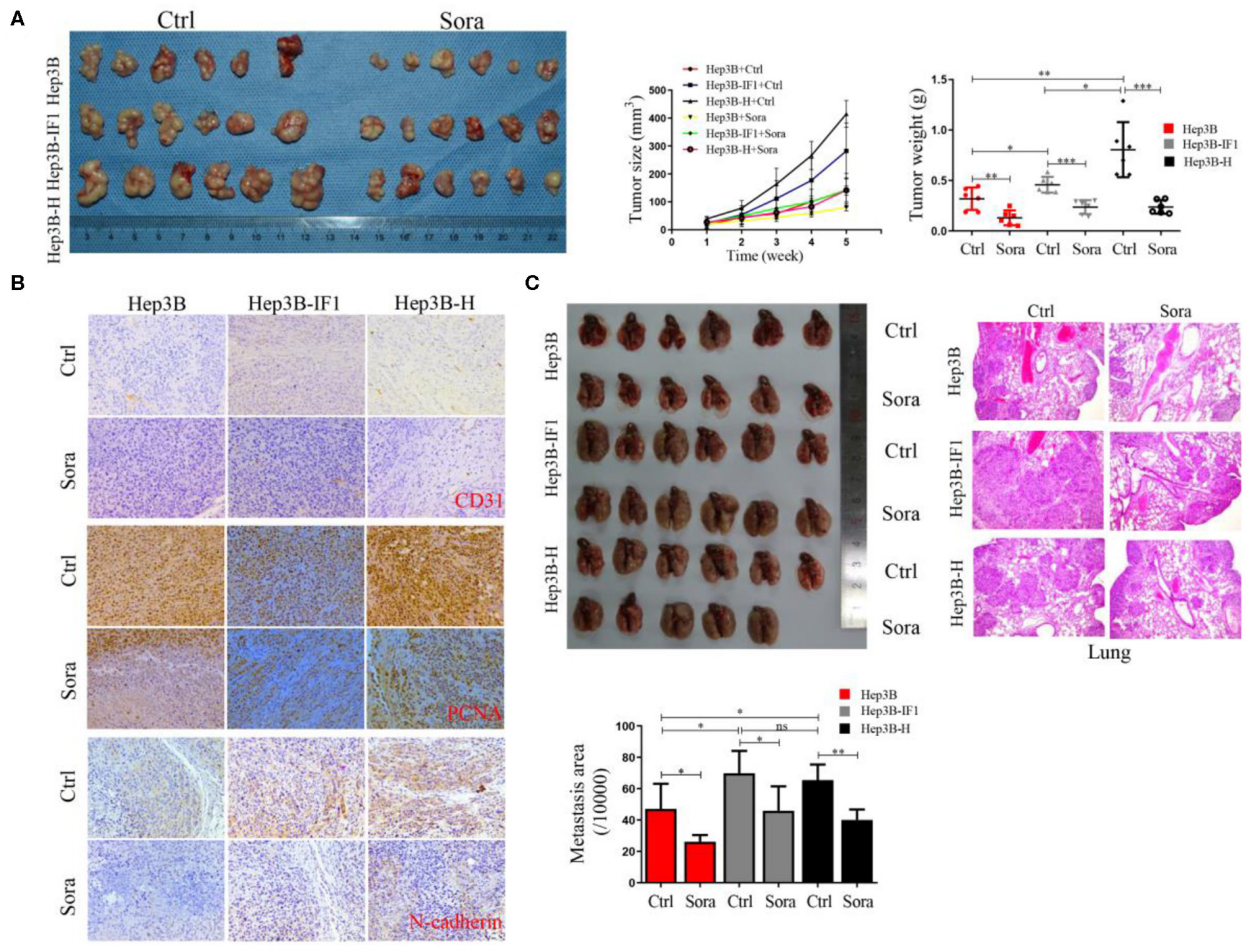
IF1 decreased the sensitivity of HCC cells after insufficient RFA to sorafenib *in vivo*. **(A)** Hep3B, Hep3B-IF1, and Hep3B-H cells were inoculated subcutaneously into nude mice. Images of tumor volume were exhibited. Tumor size was measured with a caliper rule every 1 week and tumor weight was measured at the end of the experiment. The animals were sacrificed 4 weeks after sorafenib treatment. *n* = 6 per group. **(B)** Tumor sections were stained with PCNA, N-cadherin, and CD31. Representative images of the immunohistochemistry assay were shown. **(C)** Hep3B, Hep3B-IF1, and Hep3B-H cells were inoculated into nude mice via caudal vein. The animals were sacrificed 4 weeks after sorafenib treatment. Pictures of lung metastatic nodules were shown at the end of the experiment. The area of lung metastatic nodules was measured and the representative images of HE were shown. *n* = 6 or 5 per group. Data are the means ± SD. *P < 0.05, **P < 0.01, ***P < 0.001; ns, no significance.

To further determine the effect of IF1 on sorafenib suppressing HCC cells *in vivo*, Hep3B, Hep3B-IF1, and Hep3B-H cells were inoculated into nude mice. Sorafenib could inhibit the growth of HCC cells (Figure 6A). Furthermore, the inhibition of sorafenib on Hep3B-IF1 xenograft tumors was less effective than that on Hep3B xenograft tumors. However, Hep3B-H xenograft tumors were more sensitive to sorafenib compared with Hep3B xenograft tumors (Figure 6A). Hep3B-IF1 and Hep3B-H xenograft tumors exhibited more expression of N-cadherin, CD31, and PCNA relative to Hep3B xenograft tumors (Figure 6B). Sorafenib decreased the N-cadherin, CD31, and PCNA expression in xenograft tumors (Figure 6B). IF1 overexpression reversed the decreased levels of proteins caused by sorafenib (Figure 6B). Tumor growth was decreased by 59.3, 48.5, and 70.5% in Hep3B, Hep3B-IF1, and Hep3B-H, respectively after sorafenib treatment. Next, we determined the effects of IF1 on sorafenib inhibiting metastasis of HCC cells after insufficient RFA. Sorafenib could suppress lung metastasis of HCC cells. The inhibition of sorafenib on Hep3B-H lung metastasis was less effective than that on Hep3B, but overexpression of IF1 could decrease the sensitivity of Hep3B cells to sorafenib (Figure 6C). Tumor metastasis was decreased by 44.4, 34.3, and 38.7% in Hep3B, Hep3B-IF1, and Hep3B-H, respectively, after sorafenib treatment. The difference was further confirmed by the histopathological analysis with HE staining (Figure 6C). No apparent changes were observed in body weight, heart, liver, spleen, lung, and kidney in mice in the experiment of tumor xenograft assay (Figure S8).

### NF-κB Activation Induced by IF1 Attenuated the Effect of Sorafenib in HCC Cells After Insufficient RFA

**Figure 7:**
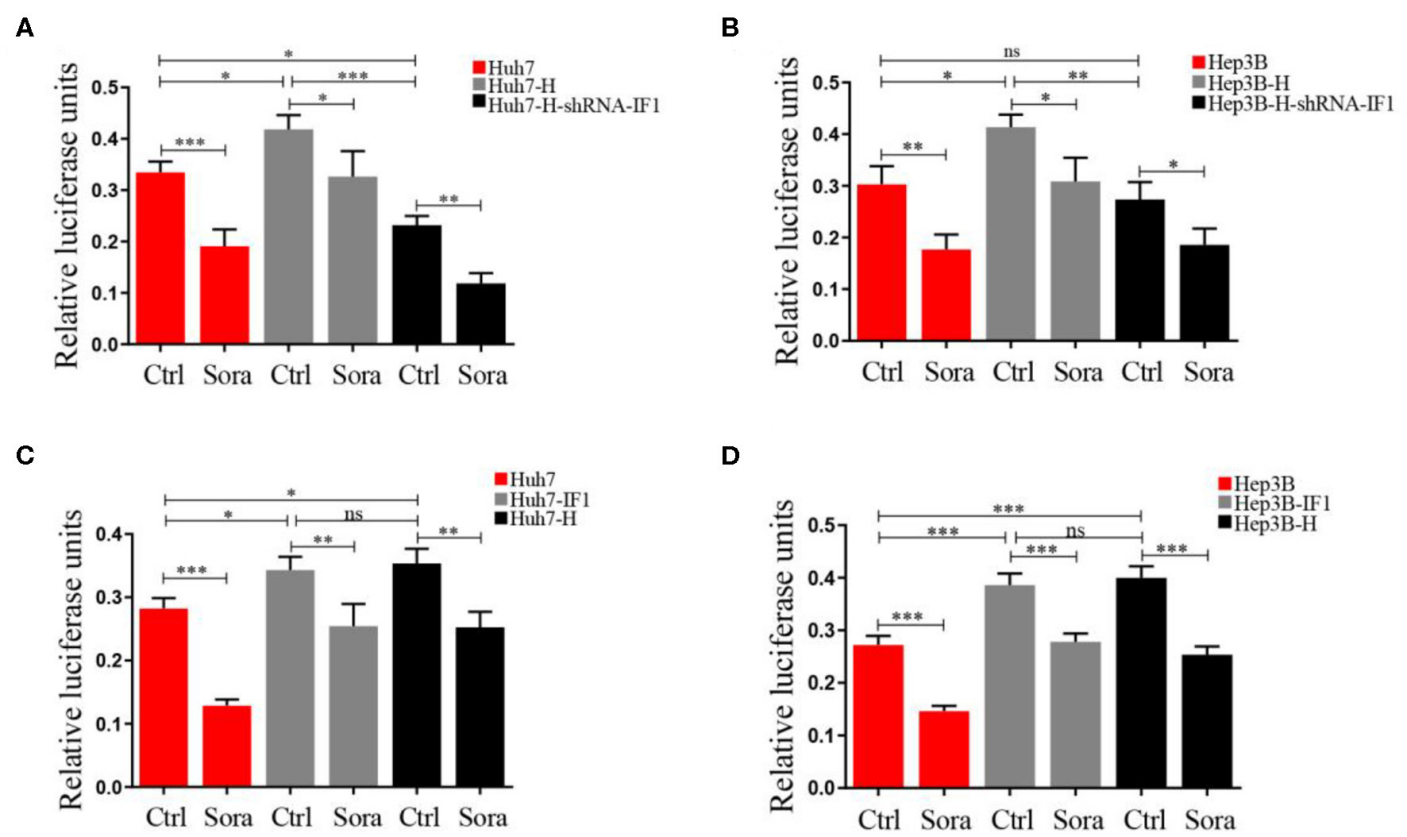
NF-κB activation induced by IF1 attenuated the effect of sorafenib on HCC cells after insufficient RFA. **(A,B)** A dual-luciferase reporter system analysis of NF-κB promoter activity after IF1 knockdown in Huh7 or Hep3B cells with or without insufficient RFA after sorafenib treatment. **(C,D)** A dual-luciferase reporter system analysis of NF-κB promoter activity after IF1 overexpression in Huh7 or Hep3B cells with or without insufficient RFA after sorafenib treatment. Data are the means ± SD of three experiments. *P < 0.05, **P < 0.01, ***P < 0.001; ns, no significance.

Activated NF-κB could promote sorafenib resistance (30), therefore we used a dual-luciferase reporter system to measure the impact of sorafenib and IF1 on NF-κB activation. Hep3B-H cell or Huh7-H exhibited higher relative luciferase activities compared with Hep3B cells or Huh7 cells (Figure 7). IF1 knockdown in Hep3B-H cells or Huh7-H reduced the relative luciferase activities, and IF1 overexpression in Hep3B or Huh7 cells increased the relative luciferase activities (Figure 7). IF1 overexpression attenuated the effect of sorafenib, and IF1 knockdown enhanced the effect (Figure 7). The expression of p-p65 was higher in Hep3B-H tumor lysates compared with Hep3B tumor lysates (Figure S9).

## Discussion

Tumor progression happens when insufficient RFA is performed, and EMT and angiogenesis may be involved in the process. EMT and angiogenesis have been linked with cellular metabolism and drug resistance (31–33). Consistent with before finding, we found that IF1 promoted the EMT and angiogenesis of HCC cells and attenuated sensitivity of sorafenib to inhibit growth and metastasis in HCC through NF-κB signal pathways.

IF1 is the physiological inhibitor of H^+^-ATP synthase and up-regulated in some prevalent carcinomas (34). IF1 could reprogram energy metabolism to an enhanced glycolysis by limiting ATP production by the H+-ATP synthase (19). IF1 expression is also an independent prognosis marker in HCC, breast cancer, and non-small cell lung cancer (22, 23, 35). EMT occurred in HCC after insufficient RFA, which enhanced the malignant potential of HCC. However, the mechanism involved in the process remains unclear. Previous study showed that IF1 could active EMT of HCC ells though activating NF-κB/Snail signaling (23). Currently, we demonstrated that IF1 expression was higher in residual tumor after insufficient RFA and meanwhile CD31 and N-cadherin expression were also up-regulated. Hep3B-H or Huh7-H showed enhanced capacity of colony formation and migration compared with Hep3B or Huh7 cells, which were consistent with the previous study, and exhibited more expression of N-cadherin and IF1. All the above results hint that IF1 may exert an important role in HCC progression after insufficient RFA. In the current study, inhibition of IF1 could attenuate the EMT process, growth and lung metastasis of Hep3B-H cells, and overexpression of IF1 could restore the EMT process of Hep3B cells. The results supported that IF1 regulated the process of EMT in HCC after insufficient RFA.

Angiogenesis participates in HCC development and progression, which is a complicated process including angiogenic factors release, angiogenic factors binding to receptors on endothelial cells, activation, migration and proliferation of endothelial cells, the extracellular matrix remodeling and tube formation (36). Incomplete RFA could accelerate angiogenesis and proliferation of residual lung carcinomas through HSP70/HIF-1α (37). Our previous study also demonstrated that residual HCC after insufficient RFA showed enhanced ability of angiogenesis through HIF-1α/VEGFA and TAECs exhibited enhanced angiogenesis after insufficient RFA r. In the present study, CM from Hep3B-H or Huh7-H cells could be more potent to TAECs migration and tube formation. Inhibition of IF1 could attenuate the angiogenesis process, induced by Hep3B-H or Huh7-H cells, and overexpression of IF1 could restore the angiogenesis process caused by Hep3B or Huh7 cells. All the data suggested that IF1 could indirectly influence TAECs migration and tube formation through some secretion factor. VM is a functional microcirculation pattern formed by aggressive cancer cells and is associated with the metastasis and poor prognosis in various cancer type, including HCC (38–41). In the present, we mixed the HCC cells and TAECs, and observed the HCC cells VM *in vitro*. IF1 inhibition could attenuate the VM process of Hep3B-H cells, and overexpression of IF1 could restore the VM process of Hep3B cells. These data demonstrated IF1 could directly influence tumor angiogenesis.

Sorafenib is the first approved oral multi-tyrosine kinase inhibitor, which is the standard first-line therapy for advanced HCC patients. Nevertheless, development of sorafenib resistance has recently caused concern on account of the high heterogeneity of individual response to sorafenib therapy (42, 43). A meta-analysis showed that combination of RFA and sorafenib exhibited no significant difference in recurrence and overall survival compared with sorafenib alone or RFA alone (44). Previous study also demonstrated that sorafenib could suppress the progression of HCC after RFA, however, whether the effect was attenuated remained unclear (17). In the current study, sorafenib could inhibit the colony and migration of HCC cells, but overexpression of IF1 could attenuated the effect of sorafenib in Hep3B or Huh7 cells, and inhibition of IF1 could improve sorafenib effect in Hep3B-H or Huh7-H cells. Similar results were also confirmed *in vivo*. TAECs also join the drug resistance, and TAECs are less sensitive to sorafenib compared to the normal counterparts (45). After inhibiting IF1 in TAECs, the sorafenib effect was more obvious. The results suggested that IF1 may be used to alter the sensitivity of HCC cells after insufficient RFA to sorafenib therapy.

Sorafenib could suppress tumor growth via NF-κB signaling pathway, but activated NF-κB mediating CD47 up-regulation promoted sorafenib resistance and its blockade synergized the effect of sorafenib in HCC (30). IF1 could activate NF-κB pathway through depending on the binding of tumor necrosis factor receptor-associated factor to NF-κB inducing kinase and the disruption of NIK association with the TRAF2-cIAP2 complex. In the present study, sorafenib could inhibit the relative luciferase activities of NF-κB, and IF1 inhibition could inhibit relative luciferase activities, but IF1 overexpression could activate the relative luciferase activities. Furthermore, IF1 overexpression attenuated the effect of sorafenib and IF1 inhibition enhanced the effect. The results demonstrate that NF-κB signaling pathway may involve in the interact effect between sorafenib and IF1.

In conclusion, our finding demonstrated that IF1 mediated the EMT and angiogenesis of HCC cells after insufficient RFA. IF1 attenuated HCC cell sensitivity to sorafenib therapy after insufficient RFA through NF-κB signaling and indicated poor disease free survival in patients with HCC after sorafenib treatment.

## Data Availability Statement

The datasets used and analyzed during the study are available from the corresponding author on reasonable request.

## Ethics Statement

The animal study was reviewed and approved by Animal Care and Use Committee of Capital Medical University.

## Author Contributions

JK, XD, and CY carried out the experiments and drafted the manuscript. SD and SW performed the animal experiment and analysis of data. LZ and WS conceived the study and coordination and helped to draft the manuscript. All authors read and approved the final manuscript.

## Conflict of Interest

The authors declare that the research was conducted in the absence of any commercial or financial relationships that could be construed as a potential conflict of interest.
