# Supplementary material for: ATPase Inhibitory Factor 1 Promotes Hepatocellular Carcinoma Progression After Insufficient Radiofrequency Ablation, and Attenuates Cell Sensitivity to Sorafenib Therapy
Source: Front Oncol. 2020 Jun 25;10:1080. doi: 10.3389/fonc.2020.01080 (PMC7330926; doi:10.3389/fonc.2020.01080)
Supplement: Supplementary file 1 [file Data_Sheet_1.pdf]

Figure S1 | The proliferation of Hep3B, Hep3B-H cells and Huh7, Huh7 cells was measured using CCK-8 assay. * * $P < 0.01$, *** $P < 0.001$.

Figure S2 | N-cadherin and IF1 expression were examined by immunofluorescence assay in Huh7 and Huh7-H cells.

Figure S3 | Colony formation assay analysis of cell proliferation after IF1 knockdown or overexpression in HCC cells after insufficient RFA with or without sorafenib therapy. (**A)** IF1 was knock down or overexpressed in Huh7-H or Huh7 cells and the colony formation ability was shown. **(B)** The effect of sorafenib on colony formation of Hep3B, Hep3B-H, Hep3B-H-shRNA-IF1 and Hep3B, Hep3B-IF1, Hep3B-H was shown.

Figure S4 | The effect of IF1 on E-cadherin expression in Hep3B, Hep3B-H, Huh7 and Huh7-H cells was measured using western blot analysis. (A) The effect of IF1 knockdown on E-cadherin expression in HCC cells. (B) The effect of IF1 overexpression on E-cadherin expression in HCC cells. * $P < 0.01$, * * $P < 0.01$, *** $P < 0.001$, ns: no significance.

Figure S5 | The effect of IF1 on lactate production in Hep3B, Hep3B-H and Huh7, Huh7-H cells was measured.

Figure S6 | Mice weight of nude mice bearing with tumor. (**A**) Hep3B, Hep3B-H, and Hep3B-H-shRNA-IF1 cells were subcutaneously injected into nude mice and mice weight was measured with a scale every 4 days. (**B**) Hep3B, Hep3B-H, and Hep3B-H-shRNA-IF1 cells were injected into nude mice via caudal vein and mice weight was measured with a scale every 4 days.

Figure S7 | IF1 increased resistance of Huh7 cells after insufficient RFA to sorafenib *in vitro.* (**A,B**) The effect of sorafenib on colony formation of Huh7, Huh7-IF1, Huh7-H and Huh7, Huh7-H, Huh7-H-shRNA-IF1 was shown. (**C**) The effect of sorafenib on migration of Huh7, Huh7-IF1, Huh7-H was shown. (**D**) The effect of sorafenib on migration of Huh7, Huh7-H, Huh7-H-shRNA-IF1 was shown.

Figure S8 | Toxicity of sorafenib on nude mice bearing with tumor. (**A**) Mice weight was measured with a scale every 1 week. (**B-F**) Heart, liver, spleen, lung, and kidney sections were stained with haematoxylin and eosin (HE). Representative images were shown.

Figure S9 | The expression of p-p65 was examined in Hep3B and Hep3B-H tumors using western blot analysis. * $P < 0.01$.

Figure S1

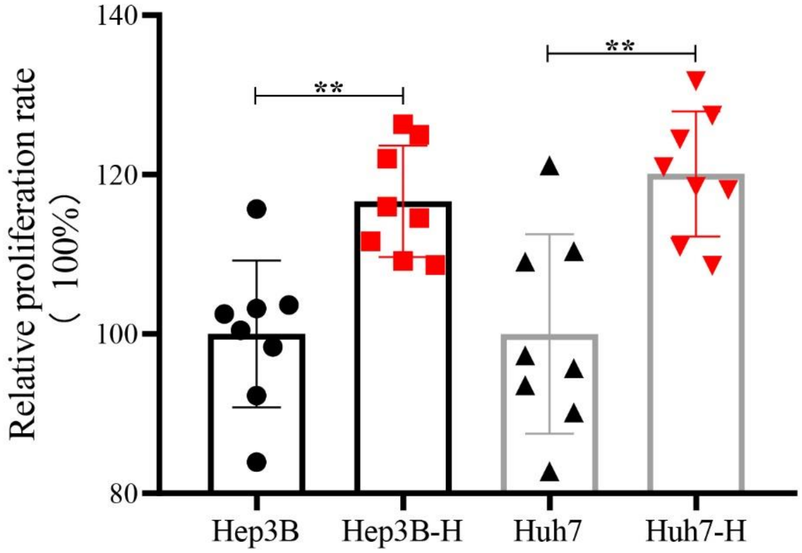
Relative proliferation rate
（100%）
140
120
100
80
**
**
Hep3B
Hep3B-H
Huh7
Huh7-H

Figure S2

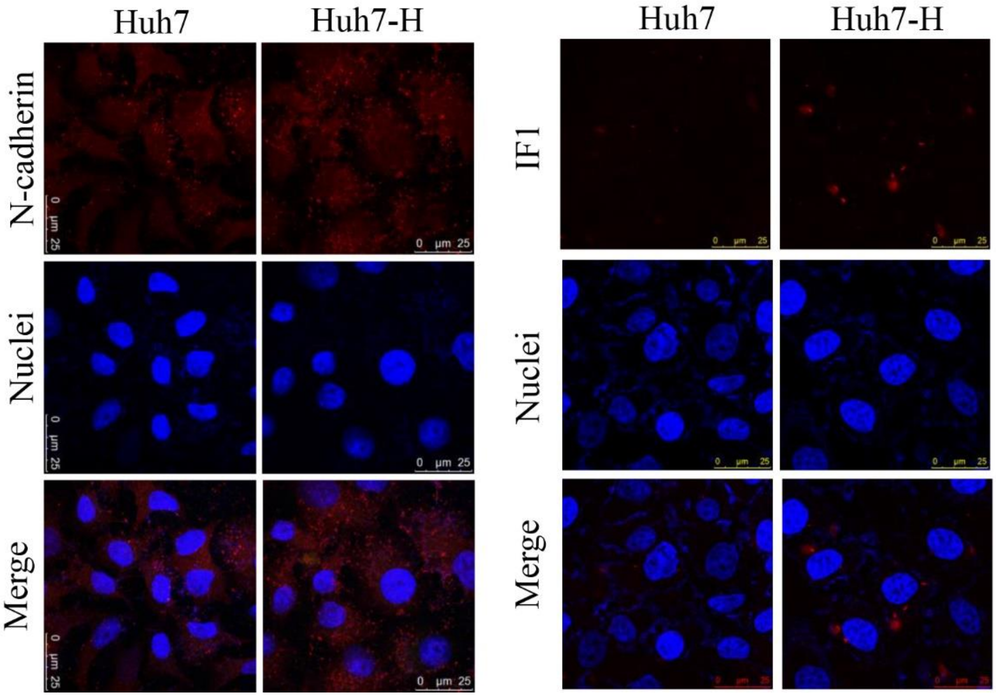
Huh7
Huh7-H
N-cadherin
Nuclei
Merge
0 µm 25
Huh7
Huh7-H
IF1
Nuclei
Merge
0 µm 25

Figure S3

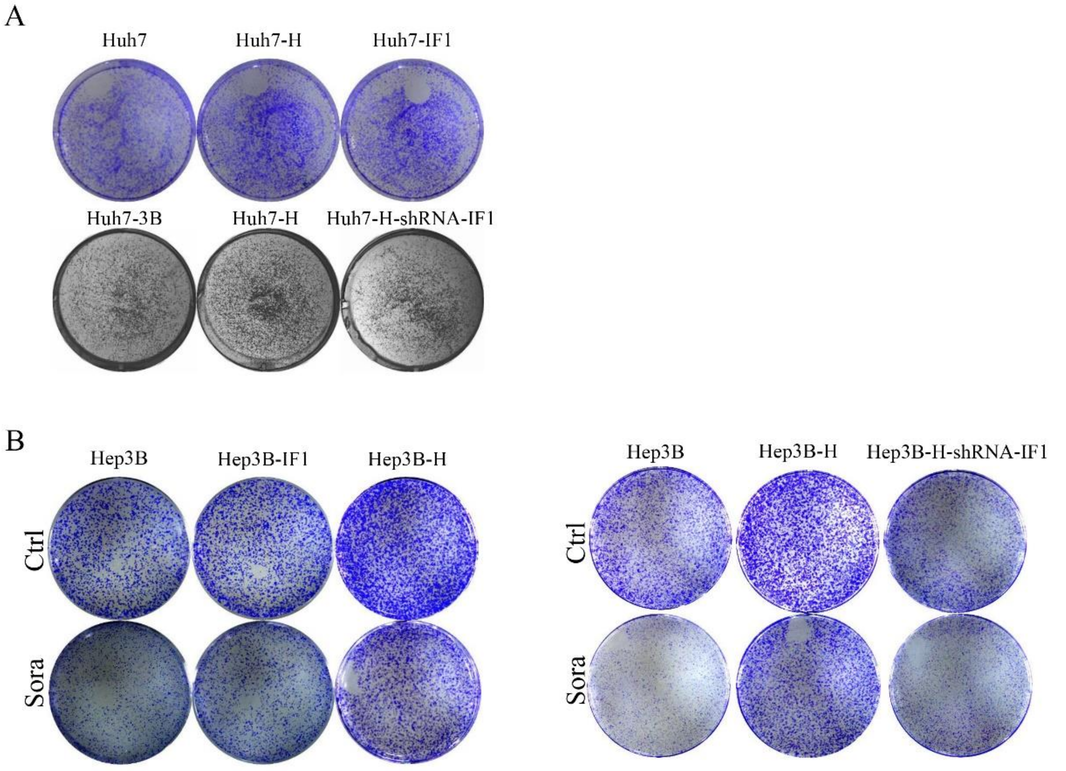
A
Huh7
Huh7-H
Huh7-IF1
Huh7-3B
Huh7-H
Huh7-H-shRNA-IF1
B
Hep3B
Hep3B-IF1
Hep3B-H
Ctrl
Sora
Hep3B
Hep3B-H
Hep3B-H-shRNA-IF1
Ctrl
Sora

Figure S4

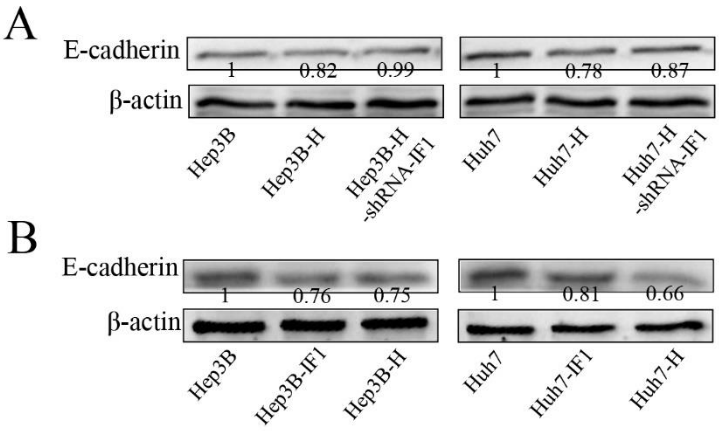

A
E-cadherin
1
0.82
0.99
1
0.78
0.87
β-actin
Hep3B
Hep3B-H
Hep3B-H -shRNA-IF1
Huh7
Huh7-H
Huh7-H -shRNA-IF1
B
E-cadherin
1
0.76
0.75
1
0.81
0.66
β-actin
Hep3B
Hep3B-IF1
Hep3B-H
Huh7
Huh7-IF1
Huh7-H

Figure S5

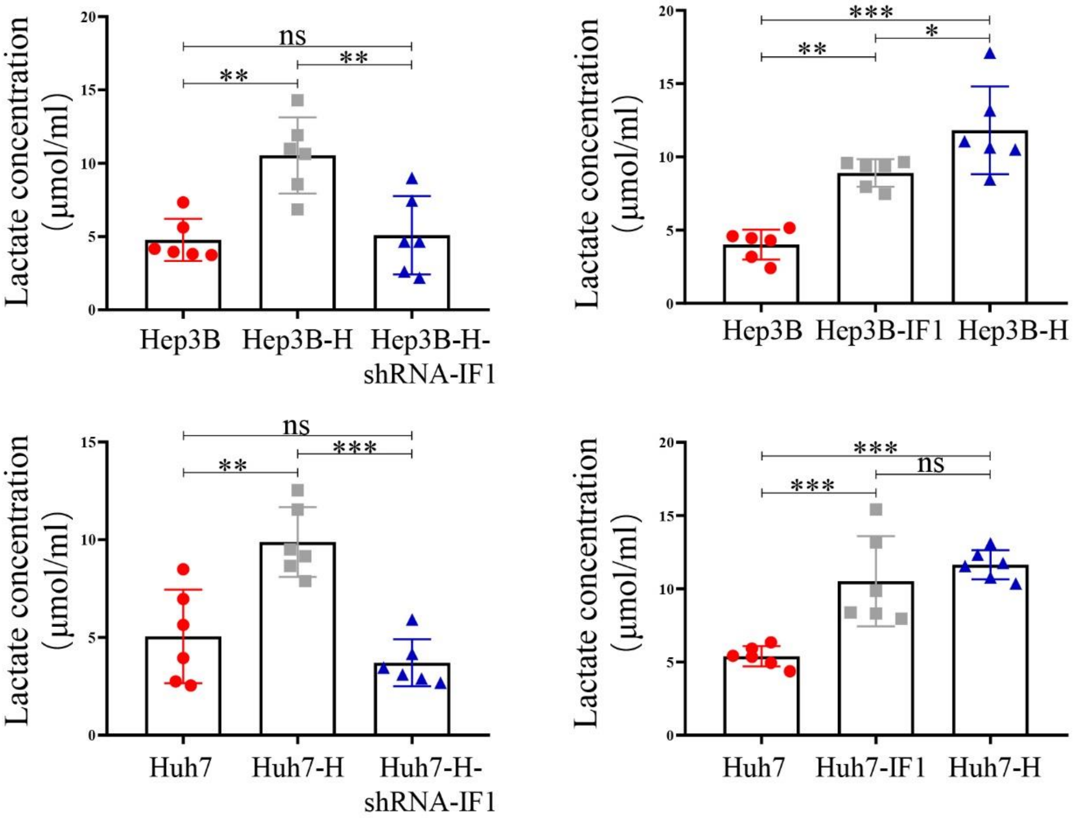

Lactate concentration (μmol/ml)
20
15
10
5
0
ns
**
**
Hep3B
Hep3B-H
Hep3B-H-shRNA-IF1
Lactate concentration (μmol/ml)
20
15
10
5
0
***
**
*
Hep3B
Hep3B-IF1
Hep3B-H
Lactate concentration (μmol/ml)
15
10
5
0
ns
**
***
Huh7
Huh7-H
Huh7-H-shRNA-IF1
Lactate concentration (μmol/ml)
20
15
10
5
0
***
***
ns
Huh7
Huh7-IF1
Huh7-H

Figure S6

A

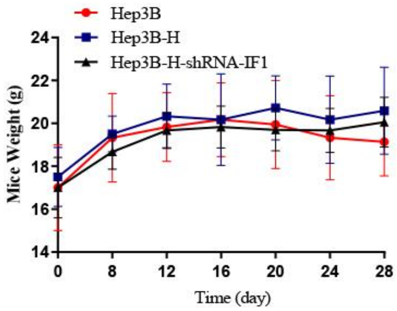
Hep3B
Hep3B-H
Hep3B-H-shRNA-IF1
Mice Weight (g)
24
22
20
18
16
14
0
8
12
16
20
24
28
Time (day)

B

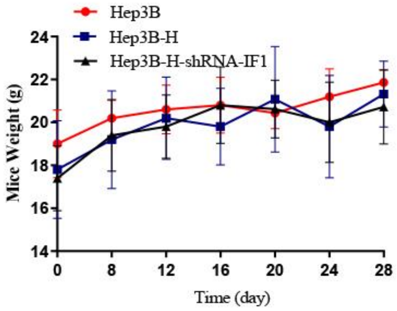
Hep3B
Hep3B-H
Hep3B-H-shRNA-IF1
Mice Weight (g)
24
22
20
18
16
14
0
8
12
16
20
24
28
Time (day)

Figure S7

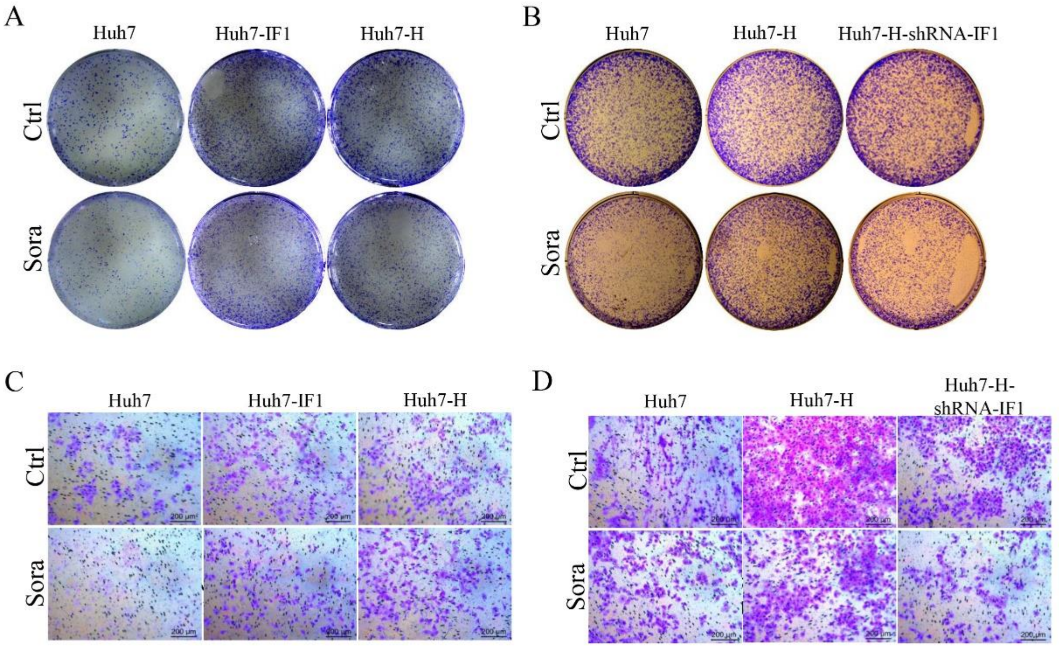
A
Huh7
Huh7-IF1
Huh7-H
Ctrl
Sora
B
Huh7
Huh7-H
Huh7-H-shRNA-IF1
Ctrl
Sora
C
Huh7
Huh7-IF1
Huh7-H
Ctrl
Sora
D
Huh7
Huh7-H
Huh7-H-shRNA-IF1
Ctrl
Sora

Figure S8

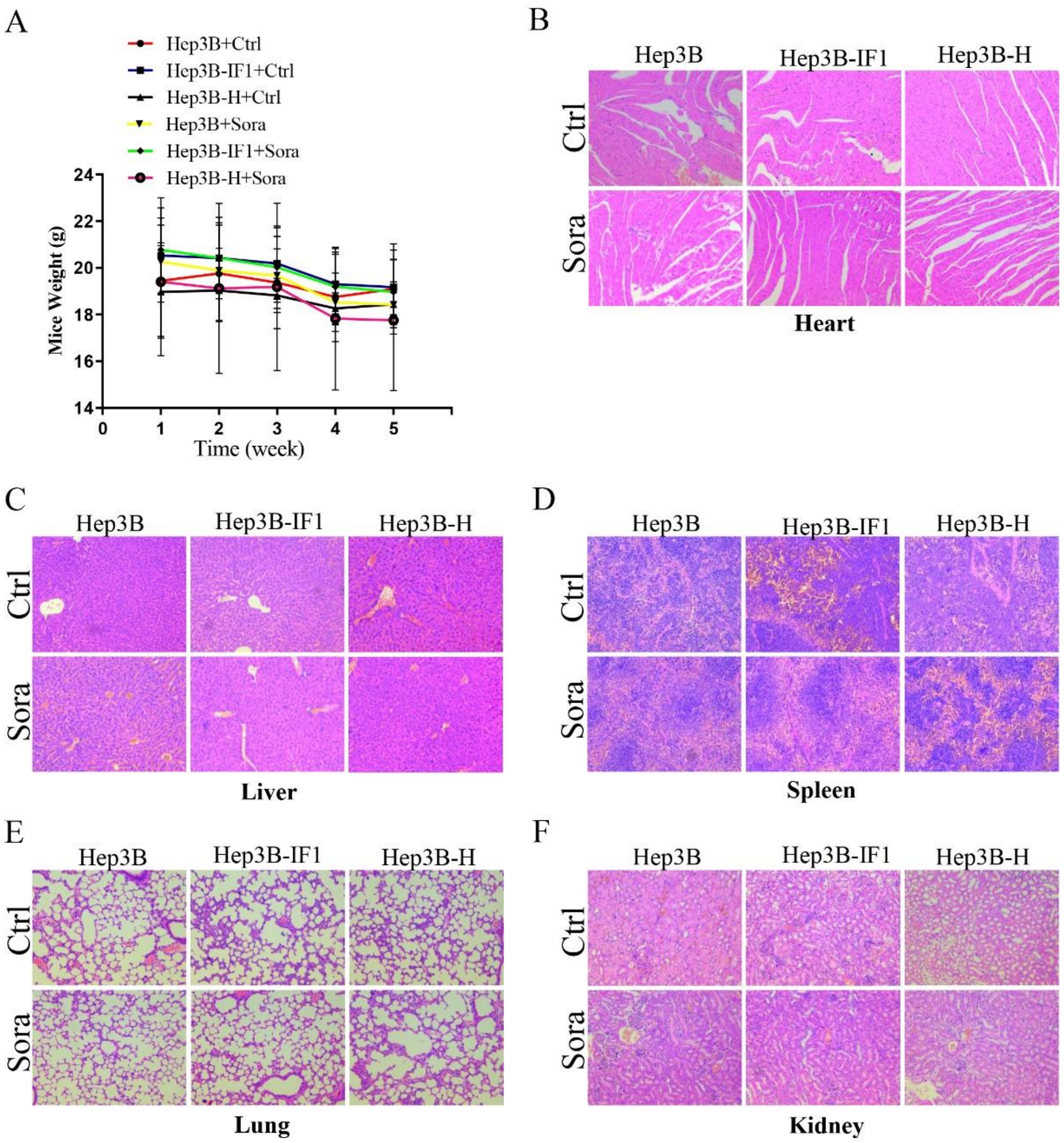
A
Hep3B+Ctrl
Hep3B-IF1+Ctrl
Hep3B-H+Ctrl
Hep3B+Sora
Hep3B-IF1+Sora
Hep3B-H+Sora
Mice Weight (g)
24
22
20
18
16
14
0
1
2
3
4
5
Time (week)
B
Hep3B
Hep3B-IF1
Hep3B-H
Ctrl
Sora
Heart
C
Hep3B
Hep3B-IF1
Hep3B-H
Ctrl
Sora
Liver
D
Hep3B
Hep3B-IF1
Hep3B-H
Ctrl
Sora
Spleen
E
Hep3B
Hep3B-IF1
Hep3B-H
Ctrl
Sora
Lung
F
Hep3B
Hep3B-IF1
Hep3B-H
Ctrl
Sora
Kidney

Figure S9

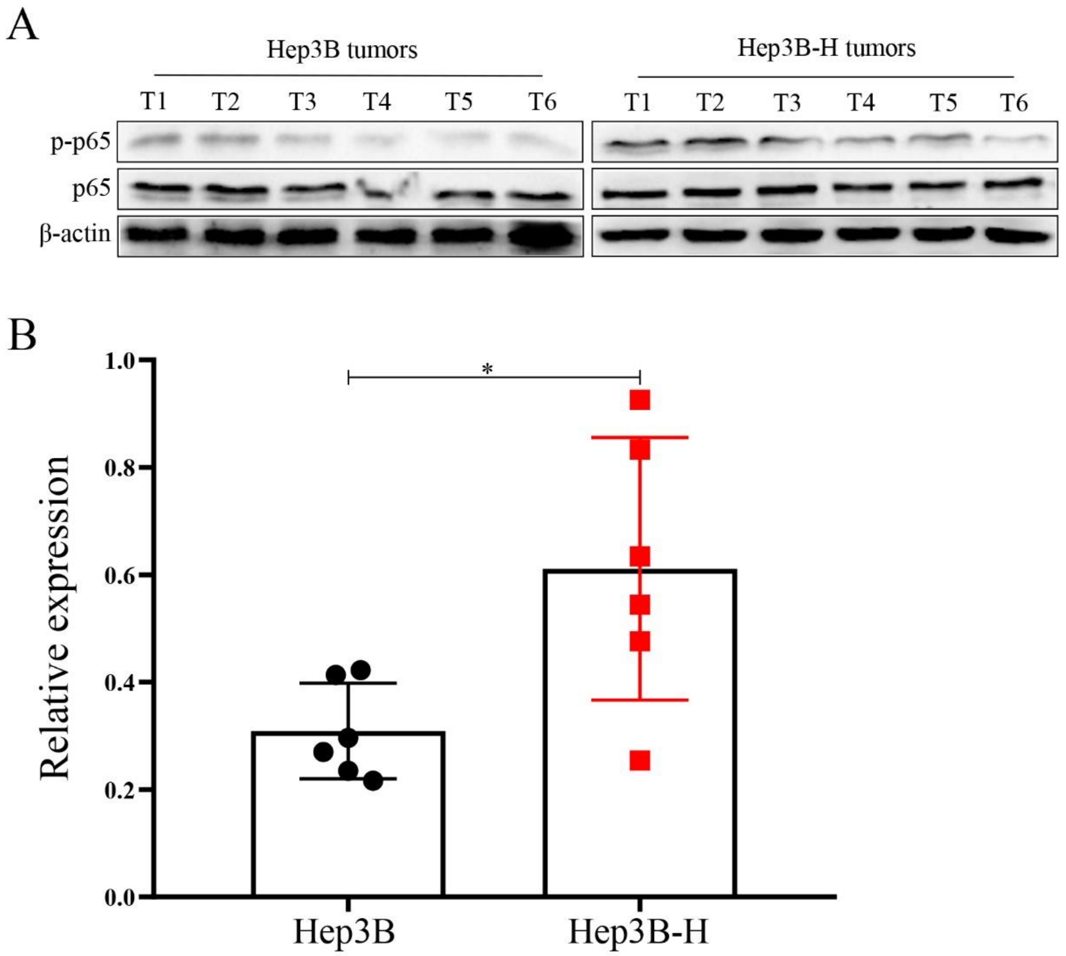
A
Hep3B tumors
Hep3B-H tumors
T1
T2
T3
T4
T5
T6
T1
T2
T3
T4
T5
T6
p-p65
p65
β-actin
B
Relative expression
1.0
0.8
0.6
0.4
0.2
0.0
*
Hep3B
Hep3B-H
